# Ictal Depth EEG and MRI Structural Evidence for Two Different Epileptogenic Networks in Mesial Temporal Lobe Epilepsy

**DOI:** 10.1371/journal.pone.0123588

**Published:** 2015-04-07

**Authors:** Negar Memarian, Sarah K. Madsen, Paul M. Macey, Itzhak Fried, Jerome Engel, Paul M. Thompson, Richard J. Staba

**Affiliations:** 1 Department of Neurology, David Geffen School of Medicine at UCLA, Los Angeles, CA, United States of America; 2 Department of Neurology, Imaging Genetics Center, Institute for Neuroimaging and Informatics, Keck School of Medicine, University of Southern California, Los Angeles, CA, United States of America; 3 UCLA School of Nursing, University of California Los Angeles, Los Angeles, CA, United States of America; 4 Department of Neurosurgery, David Geffen School of Medicine at UCLA, Los Angeles, CA, United States of America; 5 Department of Neurobiology, David Geffen School of Medicine at UCLA, Los Angeles, CA, United States of America; 6 Department of Psychiatry & Biobehavioral Sciences, David Geffen School of Medicine at UCLA, Los Angeles, CA, United States of America; Radboud University Nijmegen, NETHERLANDS

## Abstract

Hypersynchronous (HYP) and low voltage fast (LVF) activity are two separate ictal depth EEG onsets patterns often recorded in presurgical patients with MTLE. Evidence suggests the mechanisms generating HYP and LVF onset seizures are distinct, including differential involvement of hippocampal and extra-hippocampal sites. Yet the extent of extra-hippocampal structural alterations, which could support these two common seizures, is not known. In the current study, preoperative MRI from 24 patients with HYP or LVF onset seizures were analyzed to determine changes in cortical thickness and relate structural changes to spatiotemporal properties of the ictal EEG. Overall, onset and initial ipsilateral spread of HYP onset seizures involved mesial temporal structures, whereas LVF onset seizures involved mesial and lateral temporal as well as orbitofrontal cortex. MRI analysis found reduced cortical thickness correlated with longer duration of epilepsy. However, in patients with HYP onsets, the most affected areas were on the medial surface of each hemisphere, including parahippocampal regions and cingulate gyrus, whereas in patients with LVF onsets, the lateral surface of the anterior temporal lobe and orbitofrontal cortex showed the greatest effect. Most patients with HYP onset seizures were seizure-free after resective surgery, while a higher proportion of patients with LVF onset seizures had only worthwhile improvement. Our findings confirm the view that recurrent seizures cause progressive changes in cortical thickness, and provide information concerning the structural basis of two different epileptogenic networks responsible for MTLE. One, identified by HYP ictal onsets, chiefly involves hippocampus and is associated with excellent outcome after standardized anteromedial temporal resection, while the other also involves lateral temporal and orbitofrontal cortex and a seizure-free surgical outcome occurs less after this procedure. These results suggest that a more extensive tailored resection may be required for patients with the second type of MTLE.

## Introduction

Evidence suggests there are subtypes of mesial temporal lobe epilepsy (MTLE) with or without hippocampal sclerosis (HS), and some of which could have less likelihood for postsurgical seizure-freedom [1–4]. Presurgical depth electrode studies in patients with MTLE have found in some patients, the seizure onset zone (SOZ) can be localized to hippocampal structures with seizures that frequently begin with a hypersynchronous (HYP) EEG pattern [5–7]. In other cases, the SOZ is more widespread and can include polar and lateral temporal lobe, peri-sylvian, insular or orbitofrontal cortex [8,9]. These latter patients often have seizures that begin with a low voltage fast (LVF) EEG pattern [8,10,11]. HYP and LVF ictal EEG onset patterns presumably reflect different neuronal mechanisms [12,13], and if the extent of cortex supporting these two common onset seizures is different, then this could have important implications for postsurgical seizure recurrence if brain areas involved fall outside the typical margins of resection [14].

Studies have shown a link between HYP onset seizures and HS [15], specifically a classical pattern of HS with damage of dentate gyrus, CA3, CA1, and relative sparing of CA2 [16]. Furthermore, this previous study found patients with LVF onset seizures had a diffuse pattern of HS that included more damage in CA2, and was consistent with results from an MRI study that also found significant contralateral hippocampal cell loss that was not apparent in patients with HYP onset seizures [17]. While other MRI studies have shown structural damage extends beyond hippocampus in MTLE [18–20], the extent of extra-hippocampal structural abnormalities associated with HYP and LVF onset seizures is not known. Based on neuroimaging and electrophysiological data, one hypothesis is that patients with HYP onset seizures have greater damage in hippocampal and related mesial limbic structures compared to patients with LVF onset seizures who have greater structural changes in anterolateral regions of temporal and possibly frontal lobes.

In order to evaluate this hypothesis, the current study correlated depth electrode-recorded ictal EEG with changes in cortical thickness in presurgical patients who predominantly had mesial temporal LVF or HYP onset seizures. In addition, our analysis considered other clinical variables that could affect cortical thickness, particularly duration of epilepsy since previous studies found a significant effect of duration of disease on cortical thickness [21–23]. Results from our study found evidence for progressive changes in cortical thickness that had a different spatial pattern in patients with HYP onsets compared to those with LVF onset seizures.

## Materials and Methods

### Subjects & clinical depth electrode evaluation

Subjects for this retrospective study included patients with drug-resistant focal seizures of suspected temporal lobe origin who were candidates for epilepsy surgery, but required diagnostic depth electrode studies because results from non-invasive tests were inconclusive (S1 Table). For each patient, continuous depth EEG (sampled at 200Hz; bandpass 0.1–70 Hz) was recorded for a period of 1–2 weeks from eight to 14 seven-contact clinical electrodes (∅ 1.3 mm, contact length 1.5 mm; AdTech Medical Instruments, Racine, WI) that were surgically implanted orthogonal to the lateral surface of temporal bone. Depth electrodes were targeted bilaterally that regularly included amygdala, anterior, middle, and posterior regions of hippocampus, entorhinal cortex, posterior parahippocampal gyrus, and orbitofrontal cortex. Post-implant CT registered with pre-implant MRI was used to locate the position of each electrode contact with respect to anatomical brain area. All subjects provided verbal and written consent prior to participation in this study, which were recorded and stored according to the research protocol approved by the Medical Institutional Review Board in the UCLA Office for Protection of Research Subjects.

### Analysis of depth electrode-recorded ictal EEG

**Fig 1:**
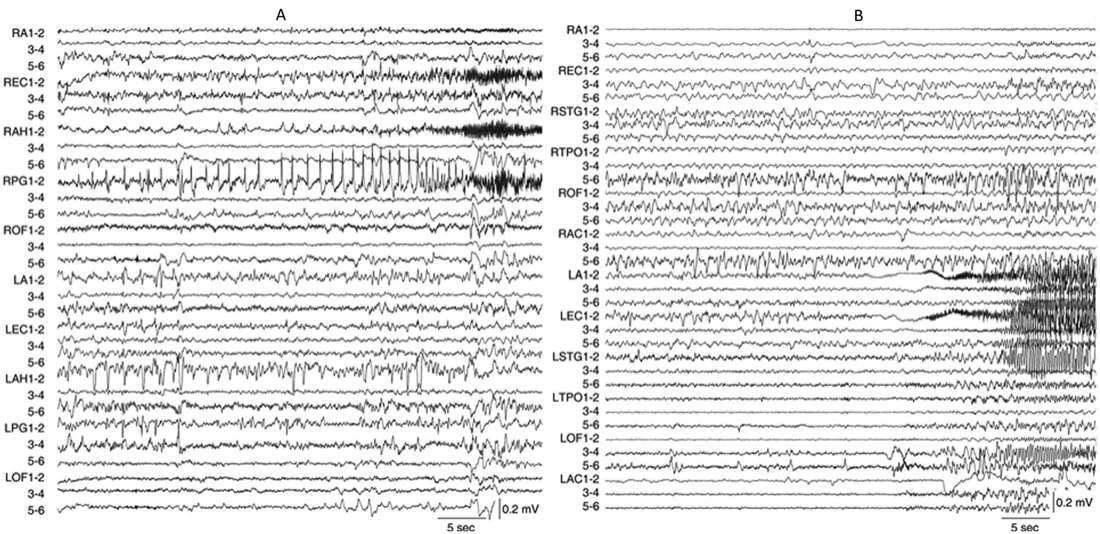
Examples of two depth electrode-recorded ictal EEG onset patterns from two different patients diagnosed with unilateral mesial temporal lobe epilepsy. (A) Depth EEG recording displayed in a bipolar montage of a focal hypersynchronous ictal onset pattern consisting of low frequency (<2 Hz), high amplitude spikes in RPG1-2 that precedes the spread to RAH1-2 and REC1-2. This seizure spread to LPG1-2 and LAH1-2 1 min 21 sec after ictal onset (not shown). Total seizure duration was 2 min 56 sec. (B) Depth EEG recording of a low voltage fast ictal onset pattern, which compared to preceding EEG baseline, begins with low amplitude, high frequency (~33 Hz) activity nearly simultaneously in LEC1-2 and LA1-2. Note ictal activity appears in LOF3-4 and 5–6 within 5 sec and in RA1-2 11 sec after ictal onset. Total seizure duration was 1 min 10 sec. Depth electrodes labels and anatomical location as follows: R (right)/L (left) A (amygdala), AC (anterior cingulate), AH (anterior hippocampus), EC (entorhinal cortex), OF (orbitofrontal cortex), PG (posterior parahippocampal gyrus), STG (superior temporal gyrus), and TPO (temporal-parietal-occipital junction); numbers refer to electrode contacts 1 (distal, medial surface) to 6 (proximal, lateral surface).

Depth EEG recordings of multiple independent spontaneous seizures were obtained from 31 patients and manually reviewed using a bipolar montage (10 mm/sec; bandpass 0.1–70 Hz). Using the UCLA Seizure Disorders Center attending neurologist’s notes on the site(s) and time of ictal EEG onset, a HYP ictal onset pattern was identified by a series of high amplitude, low frequency (≤2Hz) spike or spike-and-wave discharges that lasted 5 sec or longer (Fig 1A) [6,16]. A LVF ictal onset pattern was identified by the appearance of low amplitude, high frequency (≥10Hz) activity that occurred with or without transient EEG spike and/or slow wave (Fig 1B) [11,24,25]. Other ictal EEG onset patterns were observed less frequently in these patients, including high frequency (>13Hz) poly-spike and wave activity and rhythmic sharp wave pattern <13Hz that resembled ictal EEG onset patterns described in another study [26].

Patients were assigned to HYP or LVF seizure onset groups if three-fourths or more of a patient’s total seizures consisted of a single seizure onset type. Twenty-four patients and 110 of 118 (93%) seizures began with a HYP or LVF seizure onset pattern, while the remaining eight seizures began with poly-spike and wave or rhythmic sharp wave patterns (S2 Table). None of the 24 patients had both HYP and LVF onset seizures. The other seven patients (22 seizures) had a more heterogeneous ictal EEG onset phenotype, which will be the basis for a separate study.

In addition to identifying the SOZ, defined as the location of electrode contacts where ictal EEG activity first appeared, for each seizure the following was computed: the number of electrode contacts in the SOZ; the number of contacts, location, and delay in seconds from the SOZ to first appearance of ictal EEG activity at other recording sites in the ipsilateral and contralateral hemisphere (S1 and S2 Figs); and seizure duration was measured as the difference in time between EEG onset and termination, the latter time identified by the complete resolution of the paroxysmal, rhythmic ictal EEG pattern to a post-ictal EEG pattern [27]. In order to evaluate the spatial distribution of the site(s) of seizure onset and spread between and within each ictal onset pattern and patient, a TwoStep cluster analysis was carried out using the Akiake information criterion (SPSS, IBM Corp., Armonk, NY) [28,29]. For each seizure site(s) of onset and initial spread were represented as a one-dimensional array of binary values (1 = yes, 0 = no) for all electrode/anatomical sites sampled and used as the only input for the cluster analysis. The silhouette method was used for cluster validation, where values of +1 or -1 indicate appropriately or poorly clustered data respectively, while a value of 0 or near 0 indicates the presence of two natural clusters [30]. Paired t-tests or Mann-Whitney U tests for data not conforming to the requirement for normality were used for analysis of continuous variables, and chi-squared and exact contingency tests for analysis of categorical data where a p-value reflects the probability of finding a frequency distribution equal to or smaller than the observed distribution.

### MRI protocol

Patient preoperative MRI scans were acquired using a 1.5T Siemens Sonata full-body scanner with head coil (Siemens Medical Solutions USA, Malvern, PA, U.S.A.). Each three-dimensional T1-weighted image series was acquired in the axial plane using an MP-RAGE sequence (256×256×124 matrix, 1mm isotropic voxels). MRI scans using the same protocol, but a different 1.5T Siemens Sonata scanner, were acquired from twenty-four age- (35 ± 12.7 years) and sex-matched controls without a history of epilepsy or neurological disease at the time of this study. Results from an analysis of two subjects imaged on each scanner found a 2.7% mean error in cortical GM thickness between scanners, although differences in GM thickness due to MRI scanners were not significant (left hemisphere, p = 0.33; right hemisphere, p = 0.34).

### Analysis of MRI GM thickness

MRI analysis used a semi-automated cortical pattern matching technique combined with standardized manual tracing protocol carried out by investigators who were blinded to the identity of the subjects [31]. Briefly, the first stage involved MRI preprocessing steps of reorientation, resampling, RF correction, removal of meninges and skull, and then brain tissue excluding brain stem and cerebellum was manually masked. Masked brains images were registered to the ICBM-53 brain template with a linear 9 degrees of freedom, least square parameter (lsq9) transformation, which includes three rotations, three translations, and three rescalings in the x, y, and z dimensions. The second stage consisted of matching cortical features across individual MRI based on sulcal lines that were manually traced on the lateral and medial surfaces of three-dimensional reconstruction of each subject’s hemisphere to align cortical gyral patterns, reduce anatomical variance across subjects, and increase power to detect group differences [32]. Intra-class correlation analysis indicated strong inter-rater reliability (r = 0.94) using the manual sulcal tracing protocol on six MRI datasets [33]. The final stage, statistical comparisons of GM thickness, was carried out using an averaged GM thickness map for each ictal onset group and corresponding control group [20,34]. GM thickness was measured as the three-dimensional distance between the white-gray matter boundary in the brain tissue volume and cortical surface-cerebral spinal fluid boundary. A generalized linear model statistically evaluated the interaction between GM thickness and ictal EEG onset pattern, as well as other independent clinical variables, after controlling for age and sex at each cortical surface point, and corresponding probability or P-values were depicted on cortical surface map. Beta maps of the regression coefficients illustrate the spatial distribution in the linear rate of change of independent variable(s) as a function of GM thickness [34]. Mean percent change was calculated from GM thickness maps, as the difference between patients and controls divided by the average thickness in patients, multiplied by 100. Permutation tests (10^5^ iterations) were used to control for multiple comparisons across the entire cortical surface. Significance for all statistical tests was set at α = 0.05.

## Results

### Spatial distribution of SOZ and initial spread of HYP and LVF onset seizures

**Fig 2:**
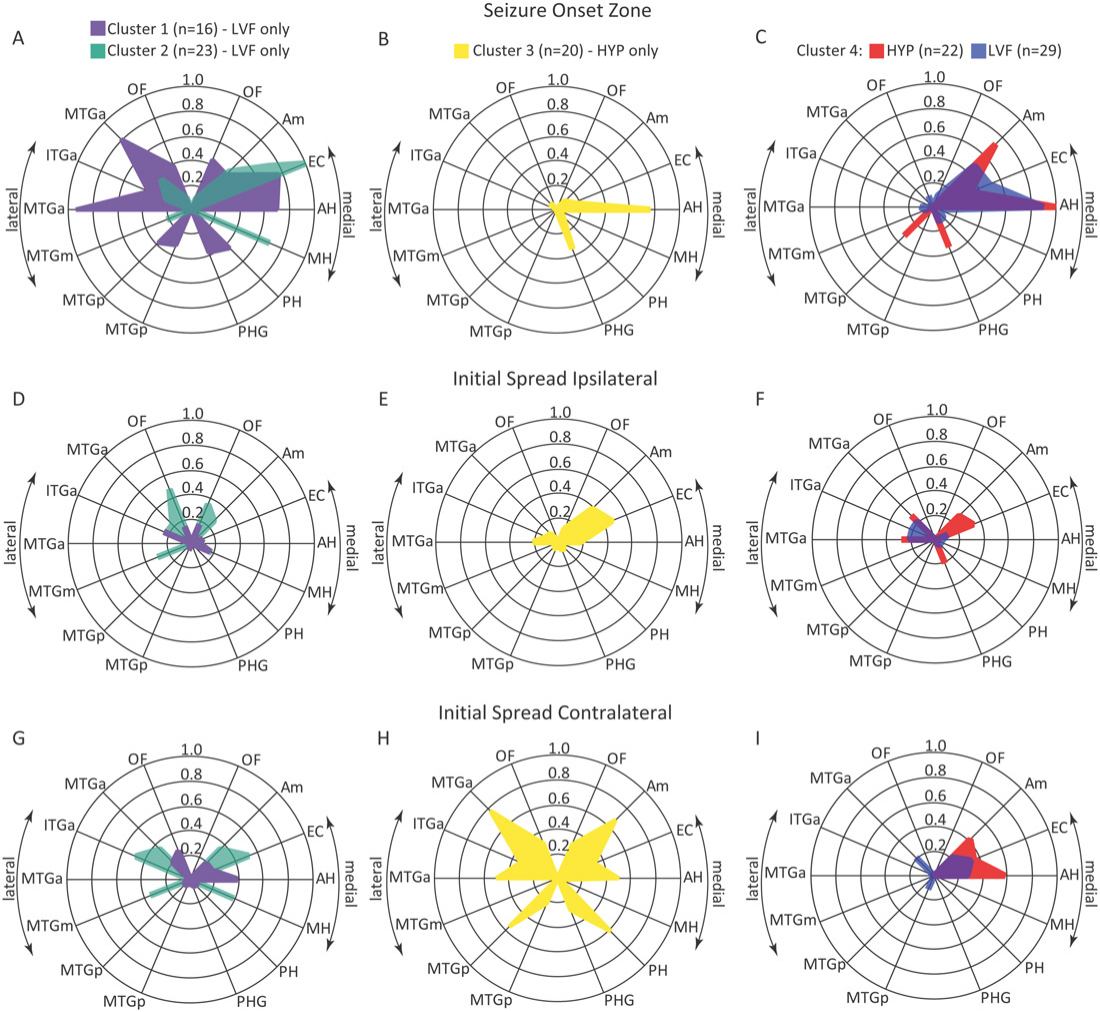
Radar plot illustrating the results of cluster analysis using anatomical /electrode sites(s) of the SOZ and initial seizure spread. Involvement of each recording site (denoted by radii on each plot) in the SOZ (top row A-C), sites of initial ipsilateral spread (middle row D-F) and initial contralateral spread (bottom row G-I) is expressed as the proportion of total seizures in each cluster (n) indicated by the concentric circles in each plot. Right and left halves of each plot depict medial and lateral aspects respectively of temporal and frontal lobe that included orbitofrontal (OF), amygdala (Am), entorhinal cortex (EC), anterior (AH), middle (MH) and posterior hippocampus (PH), parahippocampal gyrus (PHG), and anterior (a), middle (m), and posterior (p) regions of inferior (ITG) and middle temporal gyri (MTG).

Manual review of depth electrode-recorded seizures found 10 patients who consistently had unilateral HYP onset seizures (Fig 1A) and 14 patients with seizures that regularly began with a LVF ictal onset pattern (Fig 1B). Cluster analysis indicated site(s) of seizure onset (i.e., SOZ) and initial spread were consistent within each patient, but differences were found in the spatial distribution between HYP and LVF onset patterns. Overall, analysis found four clusters of seizures with a mean silhouette value of 0.2 (Fig 2). Eighteen patients (eight HYP, 10 LVF) had seizures that were associated with a single cluster, five patients (two HYP, three LVF) had seizures within two clusters, and one patient (LVF) had seizures among three clusters. The first two clusters consisted of LVF onset seizures only with a diffuse SOZ that involved entorhinal cortex, anterior or middle hippocampus, less frequently amygdala, and differential involvement of posterior hippocampus and parahippocampal gyrus, anterior aspects of middle and inferior temporal gyri, and orbitofrontal cortex (Fig 2A). By contrast, the third cluster contained HYP onset seizures only with a SOZ that chiefly involved anterior hippocampus and less frequently parahippocampal gyrus (Fig 2B). The fourth cluster contained LVF onset seizures that involved fewer sites in the SOZ compared to the first two clusters of this same EEG onset pattern (Fig 2C), but also contained HYP onset seizures that had a SOZ similar to the LVF onset seizures in this cluster (Fig 2C). Overall, compared to HYP onset seizures, LVF onset seizures first appeared on a significantly greater number of electrodes and were more likely to be recorded simultaneously in multiple mesial temporal lobe sites as well as lateral temporal and orbitofrontal cortex (Tables 1 and 2).

**Table 1:** Clinical and seizure statistics.

Demographic data/clinical history	Patients		Controls
	HYP, n = 10	LVF, n = 14	n = 24
Age	32±8.5	36±9.3	35±12.7
Gender (m/f)	8/2	7/7	15/9
Epilepsy duration, in years	20±6.8	23±11.6	
Family history of epilepsy	33%	35%	
History of febrile seizures	22%	28%	
History of CNS infection	33%	14%	
History of head trauma	22%	14%	
Seizure frequency per month	3.5 (1–15)	5.0 (2–16)	
MRI hippocampal atrophy	80%	71%	
**Seizures** ^**1**^			
No. of seizures per patient	4.0 (2–9)	4.5 (2–9)	0.48
No. of electrode contacts per patient, ipsilateral	31 (24–36)	36 (15–48)	0.23
No. of electrode contacts per patients, contralateral	30 (18–36)	24 (12–36)	0.38
Duration, in seconds	126 (35–396)	111 (20–430)	0.15
No. of contacts at seizure onset	2 (1–8)	4 (1–15)	<0.001
No. of contacts at ipsilateral spread	3 (0–9)	3 (3–18)	0.67
Latency of ipsilateral spread, in seconds	9 (0.5–90)	7 (0.2–98)	0.29
No. of contacts at contralateral spread	5 (1–12)	3 (1–9)	0.06
Latency of contralateral spread, in seconds	58 (5–202)	24 (0.8–62)	<0.001
Seizures that evolved to bilateral convulsive	35%	37%	0.38

Values represent mean ± standard deviation or median with minimum-maximum values in parentheses.

^1^ p-values reflect results of Mann-Whitney or Chi square test.

**Table 2:** Extent of SOZ and initial seizure spread.

Location		Temporal lobe						
	Ictal EEG Onset	Medial, 1	Medial, 2	Medial, ≥3	Medial & lateral	Lateral	Temporal & orbitofrontal	p-value
**SOZ** ^**a**^	HYP	45	36	7	12	0	0	0.0002
	LVF	15	26	15	27	1	16	
**Ipsilateral spread** ^**b**^	HYP	29	10	5	21	14	7	0.007
	LVF	10	1	0	16	13	25	
**Contralateral spread** ^**c**^	HYP	10	15	0	43	0	14	0.02
	LVF	4	13	10	25	10	7	

^a^ Values expressed as percent of total HYP (n = 42) or LVF (n = 68) onset seizures

^b^ Column labels “Medial, 1”, “Medial, 2”, and “Medail, ≥3” refer to number of medial temporal lobe structures in the SOZ and sites of initial seizure spread

^c^ Percentages for HYP or LVF onset seizures do not sum to 100% since not all seizures spread from the SOZ to other ipsilateral or contralateral recording sites.

With respect to the SOZ and sites of initial spread, there was no difference in delay of ipsilateral spread between HYP and LVF onset seizures (median, 9 vs. 7 sec; Table 1). For LVF onset seizures that did not involve orbitofrontal cortex or lateral temporal lobe in the SOZ, up to 48% initially spread to these ipsilateral regions (e.g., clusters 2 and 4 in Fig 2D and 2F respectively; S3 Table). Many of the HYP onset seizures beginning in hippocampus spread to entorhinal cortex and amygdala, but rarely to orbitofrontal cortex (Fig 2E and 2F), which was consistent with overall differences in sites of ipsilateral spread between LVF and HYP onset seizures (Table 2). Results of initial spread to the contralateral hemisphere found HYP onset seizures appeared after a significantly longer delay than LVF onset seizures (median, 58 vs. 24 sec; Table 1). In many cases, both types of EEG onset seizures first spread to homotopic sites contralateral to the SOZ (e.g., clusters two and four in Fig 2G and 2I respectively). However, some LVF onset seizures with a diffuse SOZ had limited spread to contralateral anterior hippocampus and orbitofrontal cortex (cluster one in Fig 2G), whereas HYP onset seizures with a focal SOZ in hippocampus spread widely in contralateral medial and lateral temporal lobe and in some cases orbitofrontal cortex (cluster three in Fig 2H).

A low voltage fast electrographic pattern was commonly recorded at sites of seizure spread in patients with HYP or LVF onset seizures (60 and 47% respectively), but rhythmic sharp wave (28 and 40%) and poly-spike and wave patterns (12 and 13%) were also observed. There was no significant difference in proportion of electrographic patterns at sites of initial seizure spread between clusters of HYP and LVF onset seizures (p = 0.12).

### Changes in cortical thickness between HYP and LVF onset seizure groups

**Fig 3:**
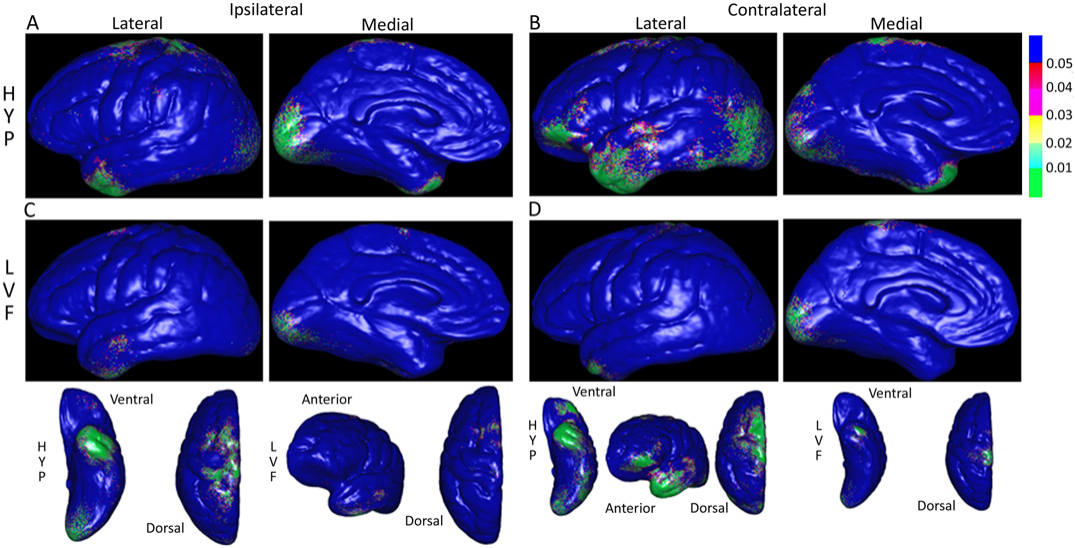
Color-coded probability or P-maps depicting the spatial pattern of significantly reduced gray matter thickness in patients with respect to controls. Lateral and medial views of cerebral hemisphere ipsilateral (A & C) and contralateral (B & D) to the SOZ in patients with hypersynchronous (HYP, top row) versus patients with low voltage fast (LVF, bottom row) onset seizures. P-maps in bottom row are the same as in rows above, but reoriented to more clearly show gray matter thickness changes on ventral, dorsal, and anterior aspects. In upper right, P-values scaled such that areas shaded green correspond to P<0.01 and blue P>0.05 (not significant).

Overall, patients had reduced cortical thickness with respect to control subjects (ipsilateral p = 0.0001; contralateral p = 0.0009). The small sample size precluded meaningful comparisons between seizure clusters and instead was carried out at the level of ictal EEG onset pattern. Both HYP and LVF onset groups had significantly reduced cortical thickness that was widely distributed in areas ipsilateral and contralateral to the SOZ (Fig 3). In each group, cortical thickness changes were located primarily in superior, middle, and inferior frontal gyri, superior aspects of pre- and post-central gyri, superior parietal gyrus, lateral and medial surfaces of occipital lobe, and anterior and lateral regions of the temporal lobe. Compared to respective controls, a greater amount of GM loss was found in the HYP than LVF seizure onset group, but the spatial pattern of loss was similar between the two ictal EEG onset groups (S3 Fig).

**Fig 4:**
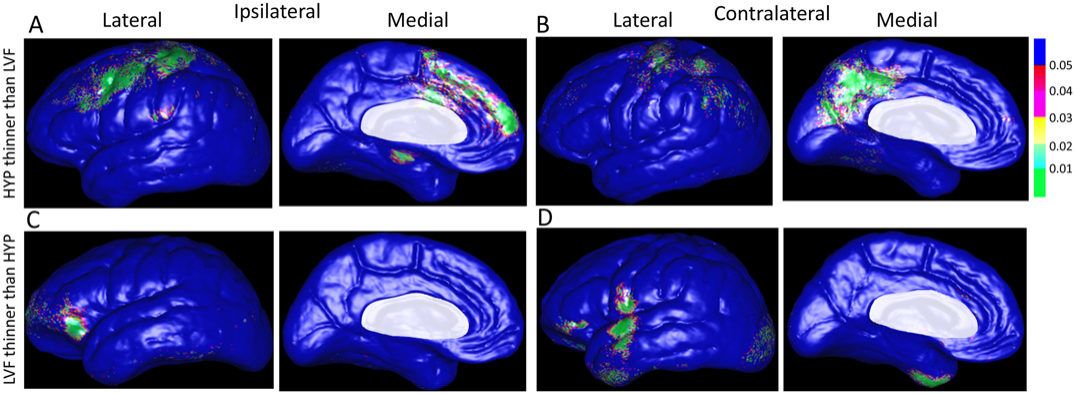
Color-coded P-maps of the lateral and medial surface of the cerebral hemisphere ipsilateral (A) and contralateral (B) to the SOZ depicting areas with a significant reduction in cortical thickness in relation to depth EEG seizure onset pattern and epilepsy duration after controlling for age and gender. Top row illustrates sites of significantly reduced gray matter thickness per year of epilepsy in patients with HYP onset seizures compared to those with LVF onset seizures, whereas bottom row reflects areas with reduced thickness in patients with LVF onset seizures with respect to those with HYP onset seizures. Permutation test correcting for multiple comparisons was significant (ipsilateral: p = 0.037; contralateral: p = 0.036). Areas masked in white (i.e., corpus callosum, diencephalon) were not included in this analysis. Color-coded p-value scale in upper right corner of the figure.

A direct comparison between HYP and LVF onset seizure groups found differences in cortical thickness that were linked with duration of epilepsy. Changes in cortical thickness alone or in combination with other clinical features were also found between patient groups, but none except epilepsy duration was significant following permutation tests. The color-coded P-maps in Fig 4 depict areas where reduced cortical thickness correlated with longer duration of epilepsy in patients with HYP onset seizures with respect to patients with LVF onsets and vice-versa. In the ipsilateral hemisphere of the HYP onset group, reduced cortical thickness per year of epilepsy was chiefly found in areas of dorsolateral and dorsomedial prefrontal cortex, caudal aspects of the frontal gyri, pre- and post-central gyri, and superior and inferior (supramarginal gyrus) parietal lobe (Fig 4A). In addition, on the medial surface locations included superior frontal gyrus, dorsal and ventral anterior cingulate gyrus, and parahippocampal gyrus. By contrast, in the LVF onset group, there were very few sites where cortical thickness correlated with epilepsy duration on the medial surface of the ipsilateral hemisphere (Fig 4C), but on the lateral surface it was mainly found in rostral and ventral aspects of frontal cortex, including inferior frontal gyrus and orbitofrontal cortex, and a few isolated areas of inferior temporal gyrus extending posteriorly to lateral aspects of fusiform gyrus.

**Fig 5:**
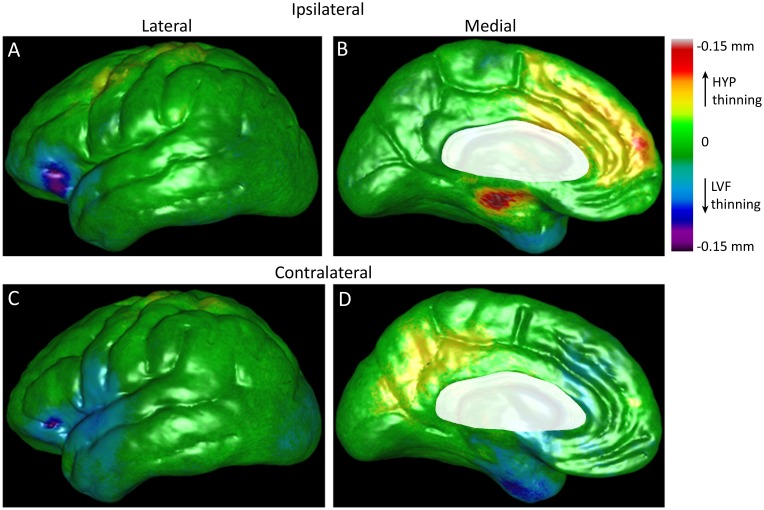
Beta maps illustrating the magnitude of the interaction between ictal EEG onset pattern and epilepsy duration on cortical gray matter thickness after controlling for age and gender. Lateral (left column) and medial surfaces (right column) of cerebral hemisphere ipsilateral (top row) and contralateral to the SOZ (bottom row). Cortical areas colored yellow, orange, and red indicate thinner GM (in mm) per year of epilepsy in patients with HYP onset seizures, whereas areas colored blue, indigo, and violet correspond to reduced GM per year of epilepsy in patients with LVF onset seizures. Shades of green depict areas of minimal or no thinning per year of disease (color-coded scale upper right corner). Permutation tests correcting for multiple comparisons were significant (ipsilateral: p = 0.037, contralateral: p = 0.037). Areas masked in white are brain areas not applicable for this analysis.

The interaction between cortical thickness and epilepsy duration, expressed as reduced GM in millimeters per year of epilepsy, is depicted on the beta maps in Fig 5. In the ipsilateral hemisphere of the HYP onset group, areas with the largest reduction in thickness per year of disease were found on the medial surface that included parahippocampal gyrus, anterior cingulate gyrus, dorsomedial prefrontal cortex, and superior frontal gyrus (Fig 5B). In the LVF onset group, reduced thickness was prominent in orbitofrontal region of inferior frontal gyrus (Fig 5A), as well as anterior temporal lobe extending towards temporopolar regions (Fig 5B).

In the contralateral hemisphere of the HYP onset group, reduced cortical thickness per year of disease was found in many of the same areas as in the ipsilateral hemisphere, including angular gyrus of inferior parietal lobe (Fig 4B). However, on the medial surface it was located more posteriorly in dorsal and ventral posterior cingulate gyrus, medial parietal lobe (precuneus), peri-striate cortex in occipital lobe, and a few isolated areas in the posterior parahippocampal gyrus. In the contralateral hemisphere of the LVF onset group, reduced GM thickness per year of epilepsy was found in the orbitofrontal region of inferior frontal gyrus, subcentral gyrus, anterior aspects of temporal gyri extending rostrally to the temporopolar area, and peri-striate cortex of lateral occipital lobe (Fig 4D). In both ictal EEG onset groups, most of these contralateral areas were also where the largest reduction in thickness per year of disease was located on the beta maps (Fig 5C and 5D).

### Postsurgical outcome

Twenty of the 24 patients had anteromedial temporal lobectomy that included resection of anterior middle and inferior temporal gyri, part of the amygdala, hippocampus, and adjacent parahippocampal gyrus [35]. Based on Engel postsurgical outcome classification [36], of the nine patients with HYP onsets who had resective surgery, outcome was Class IA in six patients (67%; one with HS, another with FCD Type Ib), IC in two patients (one with HS), and IIC in the remaining patient at an average follow up of 49 months. Of the 11 patients with LVF onset seizures who had resective surgery, outcome was Class IA in four (36%; one with HS, another with FCD Type Ib), IB in three patients (one with FCD Type Ib), IIA in one patient (FCD Type IIa), and IIIA in three patients (one with HS and FCD Type IIIa) at an average follow up of 44 months. In spite of small sample size, differences in the proportion of postsurgical outcomes between HYP and LVF onset groups were significant (p = 0.03), but not with respect to the four clusters of seizures (p = 0.39). Of the four patients who did not have resective surgery, three (one HYP, two LVF) had poor prognosis for postsurgical memory function, while the remaining patient with LVF onset seizures had poor prognosis for seizure freedom and was referred for further evaluation (S1 Table).

## Discussion

Previous depth electrode studies of MTLE found that in addition to hippocampus, sites of ictal onset and immediate spread could also involve amygdala and entorhinal cortex [7,24], posterior parahippocampal gyrus [26], temporopolar and lateral temporal cortex [8,10], insular cortex [37], or orbitofrontal cortex [38]. Furthermore, these studies indicate focal seizures frequently have onsets recorded in a single mesial temporal structure, whereas regional onset seizures involve two or more mesial temporal, lateral temporal, and/or extra–temporal structures [39]. In the present study, more than half of the seizures had a regional onset, which reflects the complexity of epilepsy that can occur in candidates for resective surgery who require depth electrode evaluation. However, analysis showed a larger proportion of seizures with HYP pattern had a focal onset in hippocampus or amygdala with subsequent spread to entorhinal cortex, and LVF onset seizures were mostly regional with onset in these same mesial structures as well as anterolateral temporal and orbitofrontal cortex. Prior work has shown entorhinal cortex plays a pivotal role in HYP and LVF onset seizures [7,26,40], which is likely due to the extensive projections this structure has with polar and anterolateral regions of temporal lobe and orbitofrontal cortex [41]. This could explain the regular involvement of these latter areas during LVF onset seizures and could be an important efferent pathway that contributed to the significantly shorter delay in contralateral spread of LVF versus HYP onset seizures [16].

Cluster analysis found groups of seizures that resemble ictal depth EEG associated with two of the several proposed subtypes of MTLE [2]. The first two clusters of LVF onset seizures in Fig 2A were similar to each other and consistent with a “mesiolateral” subtype of MTLE [10,42], while the cluster of HYP onset seizures in Fig 2B is similar to the “mesial” subtype(s) of MTLE [7]. The low silhouette value near zero suggests the presence of two clusters within the sample of seizures, yet our analysis identified a fourth cluster of seizures that had sites of onset and initial spread similar to HYP and LVF onsets seizures in the other clusters. It is evident from these data that the link between ictal EEG onset pattern and anatomical site of the SOZ is not absolute [7,40], and we cannot exclude the possibility that in some cases, the standard bilateral depth electrode placements might have missed the actual site of seizure onset. Furthermore, in contrast to the stereo-EEG approach [43], our standard bilateral approach did not regularly provide coverage of temporopolar, peri-sylvian, or insular cortex, which could help determine whether some of these seizures are associated with other subtypes of MTLE, such as “temporopolar” or “temporal plus” [44,45].

Our previous work showed patients with MTLE and HYP ictal onsets had ipsilateral hippocampal atrophy in a pattern similar to classic HS, but patients with LVF ictal onsets had a diffuse pattern of ipsilateral and contralateral hippocampal atrophy [17]. These results suggested to us that patients with HYP ictal onsets may have less extra-hippocampal damage than those with LVF ictal onsets. However, in the current study, compared to controls, patients with HYP onsets had *more* structural changes than patients with LVF onset seizures. There was no difference in cortical thickness between respective control groups that could easily explain these results. Furthermore, there was no statistical difference in epilepsy duration, seizure frequency, or percentage of secondarily generalized seizures between HYP and LVF onset groups to account for these differences. Compared to our previous hippocampal study, the greater number patients studied here could encompass a more heterogeneous sample of MTLE, including a few with “tentative” MTLE (e.g. those with poor postsurgical seizure control). Interestingly, the spatial patterns were similar between HYP and LVF onset groups and largely unrelated to the ictal depth EEG results. Since many areas with GM loss in HYP and LVF onset groups with respect to controls were the same as those found in other MRI studies of MTLE using similar [20] or different analysis techniques [46,47], it is possible the reduced thickness in Fig 3 reflects a general pattern of GM loss due to early life cerebral injury [20], long-term exposure to antiseizure drugs [48], and/or seizure-related cellular excitotoxicity [49,50]. Neuroimaging studies of MTLE have found significant interictal metabolic disturbances and ictal hyperperfusion in areas such as anterior temporal lobe and inferior frontal gyrus, as well as more remote areas of occipital cortex and regions of pre- and post-central gyri that could contribute to cellular stress and over time produce a spatial pattern of cortical damage found here [9,51].

The present study and others found a significant effect of epilepsy duration on cortical thickness that strongly suggests progressive structural, as well as epileptogenic functional changes [25,52], can occur in patients with poorly controlled seizures [21–23]. Unlike the results between patients and controls, these results showed a differential spatial pattern of reduced thickness per year of disease between HYP and LVF onset groups that was related to sites of seizure onset and initial spread. Several of the affected areas in HYP and LVF onset groups, particularly cingulate and adjacent cortices, were areas where previous resting state and interictal spike-triggered fMRI studies found reduced functional connectivity and metabolic disturbances in patients with temporal lobe epilepsy [53–55]. In addition, many of the affected areas in each ictal EEG onset group were noted in earlier non-human primate studies as key components of two architectonic and functionally-related paralimbic regions [56–58]. For example, in the HYP onset group, the most affected areas were the ipsilateral parahippocampal gyrus and anterior cingulate and contralateral posterior cingulate, which are core regions in the “hippocampocentric” axis that reflects a gradient of cortical differentiation from the hippocampal formation and extending to regions of visual and motor cortex [57]. Although most patients did not have depth electrodes targeted to cingulate or adjacent cortex, it is likely these areas were sites of seizure spread based on the high proportion of HYP onset seizures that began in hippocampus and/or parahippocampal gyrus and strong connectivity between these mesial temporal structures and cingulate gyrus [41,59–61]. By contrast, in the LVF onset group, reduced cortical thickness per year of epilepsy was mainly found bilaterally in lateral orbitofrontal cortex, anterior and lateral aspects of temporal lobe, and contralateral peri-sylvian cortex, areas that are associated with an “olfactocentric” temporopolar-insulo-orbitofrontal cortical loop [58]. While the current surface technique could not measure thickness of the insula folded inside the lateral sulcus, reduced thickness per year of disease in adjacent cortex could indicate insular damage, which we are now investigating using different techniques. Previous studies have found reduced thickness in orbitofrontal cortex of some patients with MTLE [50,62], as well as low fractional anisotropy in uncinate fasciculus [63,64], which could reflect morphological alterations that support the generation and/or spread of LVF onset seizures between mesial and extra-mesial temporal regions.

In spite of the small number of patients, a higher proportion of patients with HYP onset seizures had excellent outcome, whereas more patients with LVF onsets had only worthwhile improvement. Postsurgical MRI was not available for most patients and these data would be needed to determine if there were differences in extent of resection between the two patient groups. However, review of each patient’s neurosurgical notes suggests this was not the case and each patient had a standard anteromesial temporal resection. If this is correct, then results from the current study suggest the mesial temporal lobe plays a critical role in HYP and LVF onset seizures, yet in LVF onset seizures, regions outside the margins of resection such as peri-sylvian, lateral orbitofrontal cortex or even insula might be capable of generating seizures. Incomplete resection of epileptogenic tissue, particularly in extra-temporal cortex, is only one explanation for postsurgical seizure recurrence [14]. Consistent with evidence for progressive aspects of epileptogenicity, it is possible post-resective reorganization and deregulation of remaining limbic circuitry could support ictogenesis. Nevertheless a more extensive tailored mesial temporal resection may be indicated in patients with LVF ictal onsets and these structural changes on MRI.

## Conclusions

The burden of epilepsy, particularly decades of poorly controlled seizures, could contribute to progressive changes in cortical thickness in MTLE. The differential spatial pattern of affected cortical areas correlated with depth EEG sites of ictal onset and spread onset, which were consistent within patients for each of the HYP and LVF onset groups, and appears to correspond to two different epileptogenic networks responsible for MTLE. One, identified by HYP ictal onsets, chiefly involves hippocampus and is associated with excellent outcome after standardized anteromedial temporal resection, while the other broadly distributed network also involves lateral temporal and orbitofrontal cortex and a seizure-free surgical outcome occurs less after this procedure, which may require a more extensive tailored surgical approach.

## Supporting Information

S1 Table Patient clinical information. (DOCX)

S2 Table Ictal EEG onset patterns and number of seizures per patient. (DOCX)

S3 Table Number of seizures at onset or spread on electrodes positioned in inferior frontal/orbitofrontal gyrus per patient. (DOCX)

S1 Fig Bipolar depth electrode-recorded EEG of HYP onset seizure illustrating site(s) of ictal onset and initial ipsilateral and contralateral spread in one patient. Depth EEG recording (200 Hz sampling; bandpass 0.1–70 Hz) in bipolar montage of HYP onset seizure that begins in LA1-2, LEC1-2, and LAH1-2 (dashed vertical line, “t = 0 sec”). Initial ipsilateral spread occurred as EEG spike followed by rapid build of low voltage fast activity on LEC3-4 27 sec after onset (“+27 s”), and then spreads to the contralateral hemisphere on RA1-2 as low voltage fast activity 38 sec after ictal onset (“+38 s”). Note break in traces near middle of figure (“time gap”) corresponds to 12 sec of recording that was removed in order to show time of onset and initial spread. Abbreviations: L/R = left/right; A = amygdala, AH = anterior hippocampus, EC = entorhinal cortex, OF = orbitofrontal. Numbers corresponds to distal (1) to proximal (7) macroelectrode contacts.
(TIF)

S2 Fig Bipolar depth electrode-recorded EEG of LVF onset seizure illustrating site(s) of ictal onset and initial ipsilateral and contralateral spread in one patient. The LVF onset seizure first appears on RA1-2, RAH1-2, and RA3-4 (dashed vertical line, t = 0 s) with initial ipsilateral spread to RAH3-4 three seconds (+3 s) after onset, and then initial contralateral spread appeared on LAH1-2 as large amplitude EEG spikes 10 sec after ictal onset (+10 s). Abbreviations and numbers in labels on left same as in S1 Fig.
(TIF)

S3 Fig Color-coded maps of cerebral hemispheres that depict percent reduction in cortical thickness in patients compared to controls. Lateral and medial views of cerebral hemisphere ipsilateral (A & C) and contralateral (B & D) to the SOZ in patients with hypersynchronous (HYP, top row) versus patients with low voltage fast (LVF, middle row) onset seizures. Maps in bottom row are the same as in rows above, but reoriented to more clearly show GM thickness changes on ventral, dorsal, and anterior aspects. Note that not all areas where GM thickness was reduced were statistically significant (c.f. Fig 3 in main text).
(TIF)
