# Supplementary material for: Ictal Depth EEG and MRI Structural Evidence for Two Different Epileptogenic Networks in Mesial Temporal Lobe Epilepsy
Source: PLoS One. 2015 Apr 7;10(4):e0123588. doi: 10.1371/journal.pone.0123588 (PMC4388829; doi:10.1371/journal.pone.0123588)
Supplement: S1 Table — (DOCX) [file pone.0123588.s001.docx]

**Table S1.** Patient clinical information

|  | | | | | Non-invasive test results | | | Depth electrode results | Epilepsy surgery results | | |
| --- | --- | --- | --- | --- | --- | --- | --- | --- | --- | --- | --- |
| Pt No. | Sex / Hand | Age | Epilepsy Duration | Sz Freq | Scalp EEG | MRI | FDG-PET | Site(s) of onset | Resection | Pathology | Outcome / Follow-up |
| 340 | F / RH | 34 | 16 | 3 | L temporal | R hippocampus | mild R frontal-temporal | LAH1-2  LPG1-3  LEC1-5 | Poor prognosis for memory function with LAMTL | | |
| 341 | F / RH | 38 | 34 | 4 | L temporal | R hippocampus | R mesial temporal | RA1-4  RAH1-2 | RAMTL | Gliosis | IA / 79 |
| 344 | M / RH | 38 | 32 | 5 | Bilateral temporal | R hippocampus | R temporal-parietal | RA1-4  REC1-4  RAH1-3  RPH1-4 | RAMTL | Gliosis | IA / 38 |
| 346 | F / RH | 43 | 40 | 3 | L temporal | normal | L temporal | LEC1-2  LA1-2 | LAMTL | Gliosis | III / 47 |
| 347 | M / RH | 44 | 41 | 8 | L temporal | normal | L temporal | LEC1-4  LAH3-4  LPH3-4 | LAMTL | HS | IA / 120 |
| 350 | M / RH | 31 | 24 | 2 | Bilateral temporal | R hippocampus | normal | RAH1-2  RPH1-2 | RAMTL | HS, gliosis | IA / 36 |
| 356 | F / RH | 18 | 9 | 15 | R frontal-temporal | normal | normal | RPH1-2 | RAMTL | Gliosis | IIC / 126 |
| 360 | F / LH | 42 | 30 | 3 | Bilateral temporal | L hippocampus | Bilateral temporal | LA1-2  LEC1-2  LAH1-2 | LAMTL | Mild FCD Ib | IA / 51 |
| 364 | F / RH | 39 | 37 | 12 | R temporal-parietal | R hippocampus | mild L temporal | RPG1-2  RAH1-2 | RAMTL | Gliosis | IA / 42 |
| 368 | F / RH | 28 | 20 | 3 | R temporal | normal | normal | RAH1-2  RA1-2  REC1-2 | RAMTL | Gliosis | III / 27 |
| 371 | F / RH | 30 | 15 | 8 | L temporal-occipital | L hippocampus | normal | LAH1-2  LA1-2  LEC1-2  LPG1-2 | LAMTL | Mild FCD Ib | IB / 24 |
| 375 | F / RH | 48 | 14 | 4.5 | L temporal | L hippocampus | mild L temporal | LEC1-2  LMH1-2  LA1-2 | Poor prognosis for memory function with LAMTL | | |
| 378 | F / RH | 40 | 9 | 4 | L temporal | normal | normal | LPG1-2  LEC5-6  LAH1-2 | LAMTL | Gliosis | IA / 45 |
| 380 | M / RH | 22 | 9 | 6 | R temporal | Heterotopia R lateral ventricle | mild R temporal-parietal | REC1-4  RAH1-2  RPG1-6 | RAMTL | HS, FCD IIIA | III / 84 |
| 384 | F / RH | 25 | 20 | 3 | L frontal-temporal | R hippocampus | normal | RAH1-2 | RAMTL | Gliosis | IA / 60 |
| 385 | F / RH | 22 | 20 | 1 | L frontal-temporal | normal | normal | LA1-2  LEC1-2  LAH1-2 | LAMTL | HS, gliosis | IC / 72 |
| 388 | M / RH | 38 | 30 | 8 | Bilateral temporal | R hippocampus | normal | RAH1-2  RA1-2  REC1-2  RPG1-2 | RAMTL | Gliosis | IA / 32 |
| 394 | M / RH | 32 | 24 | 3 | R temporal-parietal | R temporal pole | normal | RPG1-7  RAH5-7 | Poor prognosis for memory function with RAMTL | | |
| 397 | F / RH | 25 | 12 | 12 | R temporal | normal | R temporal  L putamen | RAH1-2  RPG1-3 | RAMTL | Gliosis | IC / 60 |
| 401 | M / LH | 30 | 19 | 12 | Bilateral temporal | Posterior hypothalamus | L temporal-occipital | RAH1-3  REC1-3  RPG1-4 | Poor prognosis for seizure freedom, possible hypothalamic hamartoma | | |
| 415 | M / RH | 41 | 33 | 1.5 | L temporal | normal | normal | LA1-3  LEC1-3  LAH1-2 | LAMTL | Gliosis | IA / 24 |
| 418 | F / RH | 20 | 8 | 5 | L temporal | normal | mild L inferior parietal | LA1-7  LEC1-4  LMH1-4 | LAMTL | FCD IIA | II / 51 |
| 419 | F / LH | 47 | 33 | 8 | Bilateral temporal | L hippocampus | L temporal | LA1-7  LEC1-7  LAH1-7 | LAMTL | FCD Ib, gliosis | IA / 9 |
| 428 | M / LH | 46 | 6 | 8 | L frontal-temporal | L hippocampus | L temporal | LEC1-2  LMH1-2 | LAMTL | Gliosis | IB / 12 |

Epilepsy duration in years; Seizure (Sz) frequency and postsurgical follow-up in months; Outcome based on Engel classification (Engel 1987)

FDG-PET: location of hypometabolism

MRI: anatomical location and structure of reduced size on T1-weighted MRI and/or increase signal intensity on FLAIR

Abbreviations: Left (L), right (R), A=amygdala, AH=anterior hippocampus, EC=entorhinal cortex, MH=middle hippocampus, PG=parahippocampal gyrus, PH=posterior hippocampus, numbers from 1 to 7 represent most distal to proximal electrode contacts, (L/R) AMTL=anteromesial temporal lobectomy, HS=hippocampal sclerosis, FCD=focal cortical dysplasia
