# Supplementary material for: Ictal Depth EEG and MRI Structural Evidence for Two Different Epileptogenic Networks in Mesial Temporal Lobe Epilepsy
Source: PLoS One. 2015 Apr 7;10(4):e0123588. doi: 10.1371/journal.pone.0123588 (PMC4388829; doi:10.1371/journal.pone.0123588)
Supplement: S2 Table — (DOCX) [file pone.0123588.s002.docx]

**Table S2.** Ictal EEG onset patterns and number of seizures per patient
