# Supplementary material for: Ictal Depth EEG and MRI Structural Evidence for Two Different Epileptogenic Networks in Mesial Temporal Lobe Epilepsy
Source: PLoS One. 2015 Apr 7;10(4):e0123588. doi: 10.1371/journal.pone.0123588 (PMC4388829; doi:10.1371/journal.pone.0123588)
Supplement: S3 Table — (DOCX) [file pone.0123588.s003.docx]

**Table S3.** Number of seizures at onset or spread on electrodes positioned in inferior frontal gyrus/orbitofrontal cortex per patient

|  | | No. of seizures and delay in sec | | | | |
| --- | --- | --- | --- | --- | --- | --- |
| Patient ID | Ictal EEG onset pattern | Orbitofrontal electrodes | Ipsilateral | Delay | Contralateral | Delay |
| 340 | HYP | B | 3 | 7 | 4 | 12 |
| 341 | LVF | none | - |  | - |  |
| 344 | LVF | none | - |  | - |  |
| 346 | LVF | B | 0 |  | 0 |  |
| 347 | LVF | B | 2 | 0 | 0 |  |
| 350 | HYP | B | 0 |  | 0 |  |
| 356 | HYP | none | - |  | - |  |
| 360 | HYP | L | 0 |  | - |  |
| 364 | HYP | B | 0 |  | 0 |  |
| 368 | LVF | B | 1 | 0 | 0 |  |
| 371 | LVF | B | 0 |  | 0 |  |
| 375 | LVF | L | 4 | 29 | ­- |  |
| 378 | HYP | L | 0 |  | - |  |
| 380 | LVF | B | 2 | 0 | 0 |  |
| 384 | HYP | B | 0 |  | 0 |  |
| 385 | HYP | B | 0 |  | 0 |  |
| 388 | HYP | B | 0 |  | 0 |  |
| 394 | LVF | B | 3 | 0 | 0 |  |
| 397 | HYP | none | - |  | - |  |
| 401 | LVF | B | 0 |  | 0 |  |
| 415 | LVF | B | 3 | 17 | 0 |  |
| 418 | LVF | B | 8 | 11 | 0 |  |
| 419 | LVF | B | 2 | 35 | 0 |  |
| 428 | LVF | B | 0 |  | 0 |  |

HYP = Hypersynchronous, LVF = Low voltage fast

Position of orbitofrontal electrodes: B = bilateral, L = left hemisphere, R = right hemisphere

Mean delay (in seconds) computed as difference in time between first appearance of ictal discharges at orbitofrontal electrodes and ictal onset in mesial temporal lobe. A value of 0 sec indicates ictal onset appeared simultaneously on mesial temporal and orbitofrontal electrodes.
