# Supplementary figures and images for: Ictal Depth EEG and MRI Structural Evidence for Two Different Epileptogenic Networks in Mesial Temporal Lobe Epilepsy
Source: PLoS One. 2015 Apr 7;10(4):e0123588. doi: 10.1371/journal.pone.0123588 (PMC4388829; doi:10.1371/journal.pone.0123588)

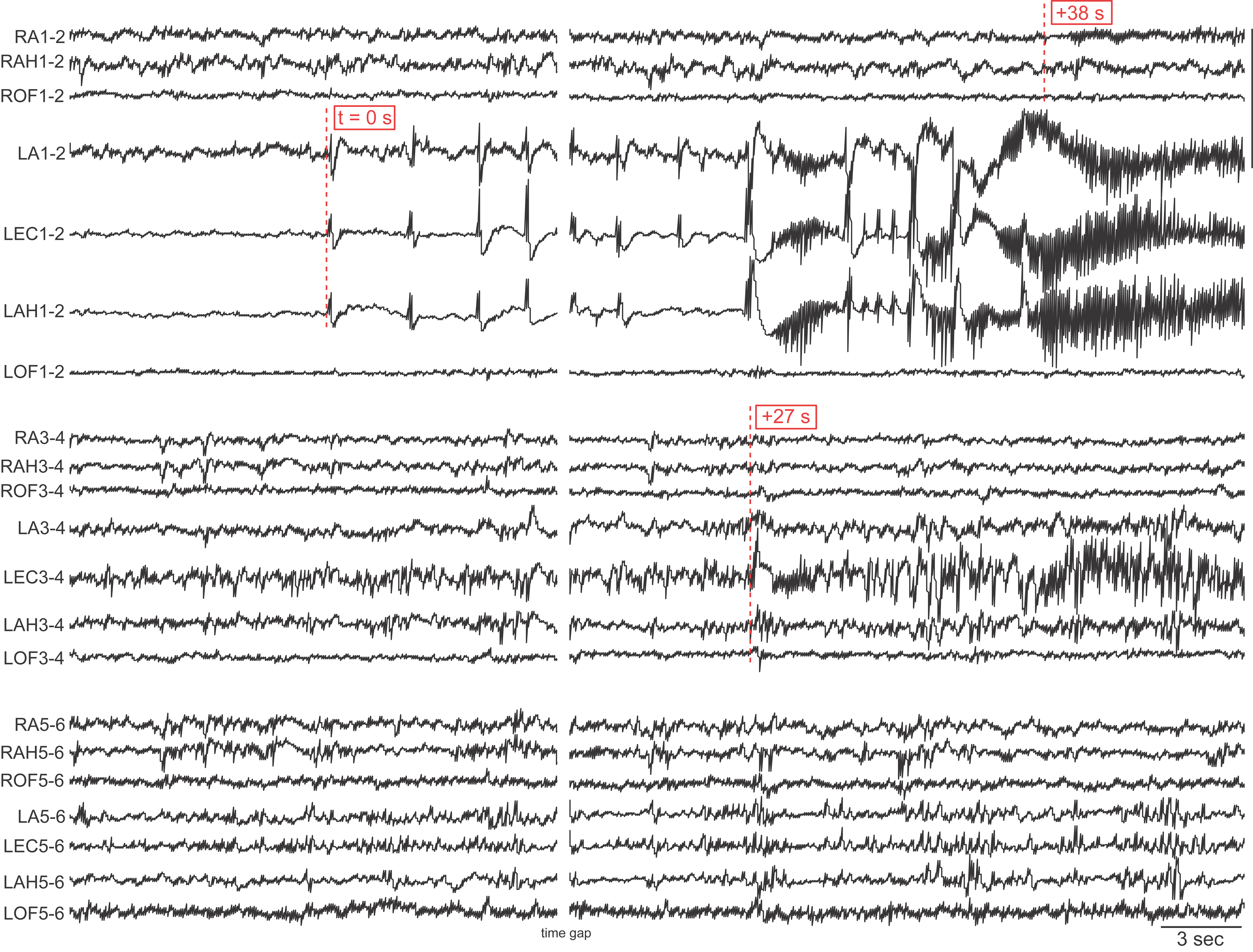

Supplement: S1 Fig — Depth EEG recording (200 Hz sampling; bandpass 0.1–70 Hz) in bipolar montage of HYP onset seizure that begins in LA1-2, LEC1-2, and LAH1-2 (dashed vertical line, “t = 0 sec”). Initial ipsilateral spread occurred as EEG spike followed by rapid build of low voltage fast activity on LEC3-4 27 sec after onset (“+27 s”), and then spreads to the contralateral hemisphere on RA1-2 as low voltage fast activity 38 sec after ictal onset (“+38 s”). Note break in traces near middle of figure (“time gap”) corresponds to 12 sec of recording that was removed in order to show time of onset and initial spread. Abbreviations: L/R = left/right; A = amygdala, AH = anterior hippocampus, EC = entorhinal cortex, OF = orbitofrontal. Numbers corresponds to distal (1) to proximal (7) macroelectrode contacts. (TIF) [file pone.0123588.s004.tif]

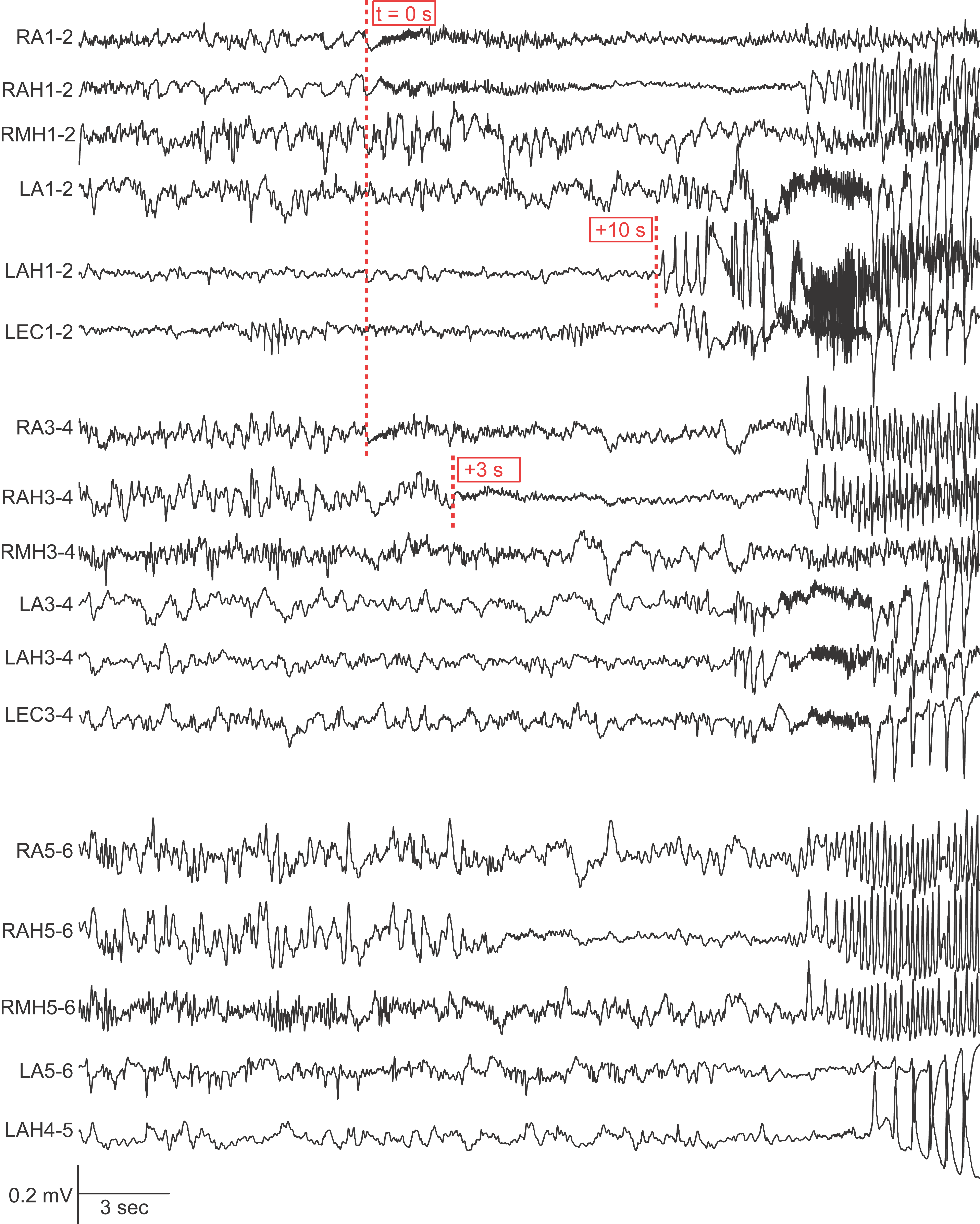

Supplement: S2 Fig — The LVF onset seizure first appears on RA1-2, RAH1-2, and RA3-4 (dashed vertical line, t = 0 s) with initial ipsilateral spread to RAH3-4 three seconds (+3 s) after onset, and then initial contralateral spread appeared on LAH1-2 as large amplitude EEG spikes 10 sec after ictal onset (+10 s). Abbreviations and numbers in labels on left same as in S1 Fig. (TIF) [file pone.0123588.s005.tif]

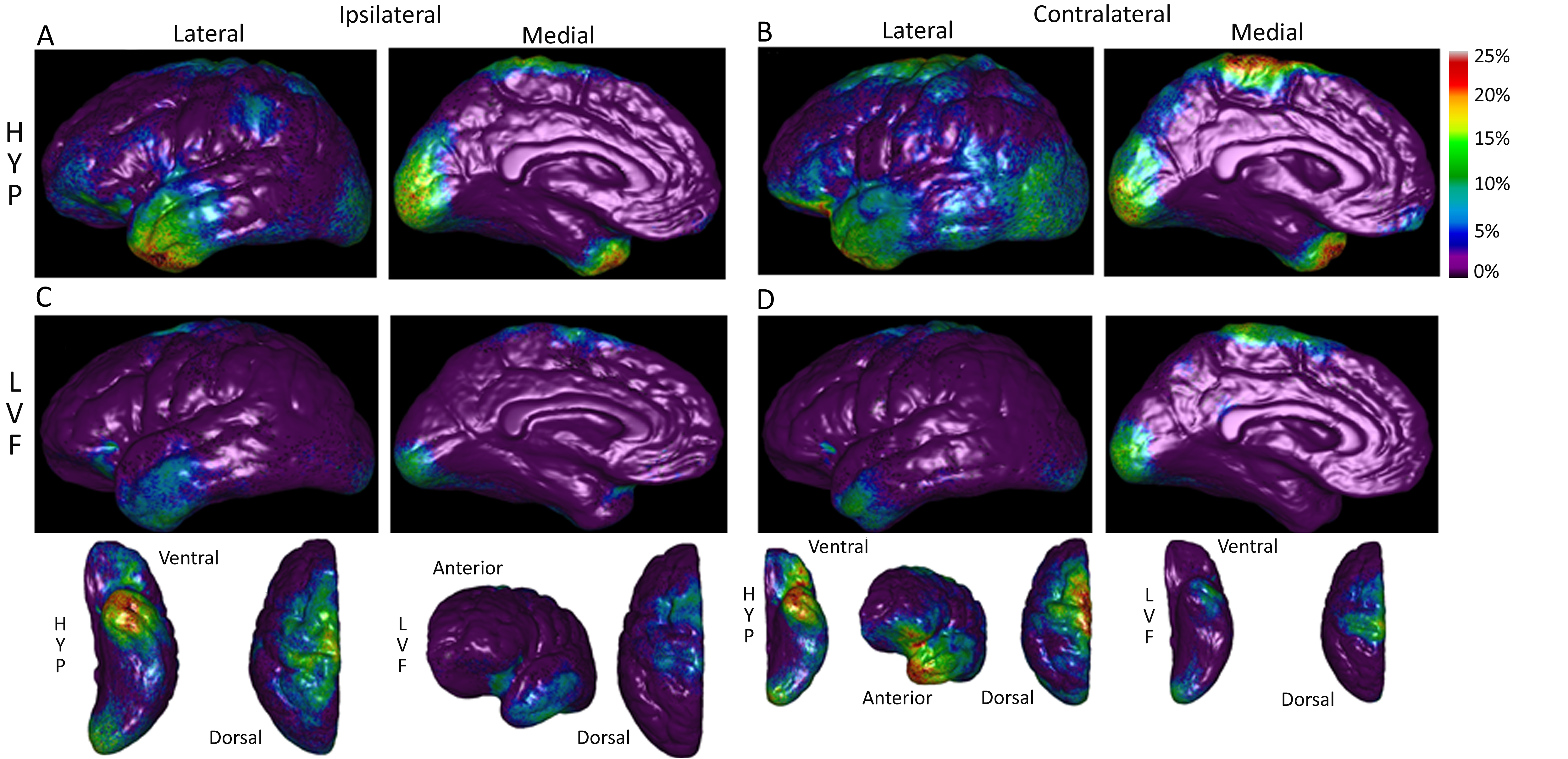

Supplement: S3 Fig — Lateral and medial views of cerebral hemisphere ipsilateral (A & C) and contralateral (B & D) to the SOZ in patients with hypersynchronous (HYP, top row) versus patients with low voltage fast (LVF, middle row) onset seizures. Maps in bottom row are the same as in rows above, but reoriented to more clearly show GM thickness changes on ventral, dorsal, and anterior aspects. Note that not all areas where GM thickness was reduced were statistically significant (c.f. Fig 3 in main text). (TIF) [file pone.0123588.s006.tif]
